# Experimental investigation on fatigue life and tensile strength of carbon fiber-reinforced PLA composites based on fused deposition modeling

**DOI:** 10.1038/s41598-023-45046-x

**Published:** 2023-10-24

**Authors:** Ehsan Kargar, Ahmad Ghasemi-Ghalebahman

**Affiliations:** Faculty of Mechanical Engineering, Semnan University, Semnan, Iran

**Keywords:** Composites, Mechanical engineering

## Abstract

Fused deposition modeling (FDM) is a widely used additive manufacturing (AM) method that offers great flexibility in fabricating complex geometries without requiring expensive equipment. However, compared to other manufacturing methods, FDM-produced parts generally exhibit lower strength and fatigue life. To overcome this limitation, researchers have explored the use of fibers and reinforcements to enhance the mechanical properties of FDM parts. Nevertheless, the performance of FDM-produced parts can be significantly affected by various manufacturing parameters, including infill density, which is a key factor in balancing time and cost. In this study, the tensile strength and fatigue life of carbon fiber-reinforced polylactic acid (PLA) composites produced by FDM were investigated by varying the infill density (50 and 75%) and raster angle (0°, 45°, and 90°). The effects of 100% filling density, raster width, and nozzle diameter on mechanical properties were also examined. The experimental results demonstrated that increasing the infill density and decreasing the raster angle can enhance the tensile strength, although the fatigue behavior was found to be more complex and dependent on the infill density. The optimal parameters for producing FDM parts with improved mechanical properties were identified based on the analysis of the tensile strength and fatigue life data. This research has yielded significant findings concerning the diverse fatigue behavior associated with the raster angle at different infill densities. Specifically, noteworthy observations reveal that a raster angle of 45 degrees at 50% infill density, and a raster angle of 0 degrees at 75% infill density, exhibited the most prolonged fatigue life. This outcome can be ascribed to the specific loading conditions and the inherent strength of the sediment layer at the critical point of stress concentration.

## Introduction

Additive manufacturing is also known as 3D printing. It has been used in the automotive and aviation industries since the early 1980s. The production of required geometries, and prototypes of high-volume industrial parts with the purpose of the trial, to optimize cost and materials, can be produced by practical use of the additive manufacturing process. FDM technology is one of the most extensive additive manufacturing methods in which parts are produced layer by layer by extruding thermoplastic materials. In FDM manufacturing, the optimal selection of parameters has a great impact on the mechanical properties of the printed part and the print quality. Polylactic acid is a biodegradable thermoplastic and one of the most common materials used in 3D printing. Although additive manufacturing has many advantages compared to other manufacturing processes, it also has a significant reduction in strength^1^. Many studies have been done in the field of pure PLA and the addition of reinforcing materials such as fibers or particles in 3D printed parts and the effect of printing parameters on mechanical properties and fatigue behavior^2–8^.

Khosravani et al.^9^ compared the samples with different grid directions, between 0 and 90 degrees, and with two print speeds of 20 mm/s and 80 mm/s under the tensile test. the tensile strength increased by decreasing the raster direction and printing speed. Heydari Rarani and colleagues^10^. Three-point bending and tensile tests of pure PLA and continuous carbon fibers reinforced with PLA were investigated, therefore an extruder was designed and built to produce composite thermoplastics with continuous carbon fibers for induction on an FDM printer. Carbon fiber-reinforced PLA had higher tensile and flexural strength than pure PLA, although the failure strain was better in pure PLA. Liu et al.^11^ compare the bending and tensile strength of pure PLA and wood, carbon fiber, ceramic, copper, and aluminum composites based on PLA and investigate the effect of + 45°/− 45° and + 0°/− 90° print angles. and the placement angle of the samples when printing for payment. Rajpurohit et al.^12^ compared the print angle of 90, 45, and 0 degrees, layer height, and raster width using the tensile test, where 90 degrees had the lowest and 0 degrees the highest tensile strength. Bembenek et al.^13^ investigated the tensile strength of PLA and PET-G material, printing temperature, layer height, and infill density were the compared parameters. which also measured the mentioned parameters by neutralizing the effect of weight on tensile strength. Brischetto et al.^14^ investigated the compressive and tensile strength behavior of PLA material. PLA samples produced by the FDM method exhibited completely different behavior compared to other methods. Hannon et al.^15^ investigated the tensile strength of PLA and high-temperature PLA with filler densities of 100, 50, and 6 in the lattice direction and three manufacturing directions x, y, and z. The remarkable result in this research is the closeness of the length increase values of 50% and 100% of the samples in the x and y directions. Naveed^16^ compared the tensile strength of two filaments made of PLA and Tough-PLA at two raster angles (45–45) and (90–0) along with three different printing speeds. Forcada et al.^17^ based their work on the fatigue life of polycarbonate made with the FDM technique; The investigated parameter in the printing of parts was the manufacturing orientation. Letcher and Waytashek^18^ made samples with 100% filling density and three Raster directions for tensile, bending, and fatigue tests with PLA material by the FDM method. Afrozeh et al.^19^ subjected samples made of PLA material to static tensile and cyclic tensile loads. Azadi et al.^20^ made samples with a 50% filling density of ABS and PLA by the FDM method. A parameter other than the material that was examined was the direction of the print. In the field of carbon fibers reinforced PLA filament, Rao et al.^21^ and Kumar et al.^22^ investigated the effect of manufacturing parameters on tensile strength.

The analysis of tensile strength and high cycle fatigue life of composite thermoplastics fabricated by the FDM method is a new field. In the present work, the tensile strength and fatigue behavior of carbon fiber-reinforced PLA were analyzed. The infill density of 50% and 75% and the raster angle of 0, 45, and 90 degrees were among the parameters investigated in the fatigue test. Also, to extract the mechanical properties of 100%, 75%, and 50% filling, raster angles 0, 45, and 90 degrees, nozzle diameter, and layer thickness were subjected to the tensile test. Data analysis was done to analyze the effect of parameters on fatigue life and tensile strength.

In both the automotive sector and the production of consumer goods, the rising demand for lightweight materials to enhance fuel efficiency and create long-lasting, robust products has opened up opportunities for using composite thermoplastics with improved tensile strength and fatigue performance. These materials offer the potential to manufacture lightweight automotive parts while maintaining safety and durability, as well as producing sports equipment, electronics, and household items with enhanced mechanical properties.

The novelty of this research is in its focus on a new and emerging field, the use of a specific composite material (carbon fiber-reinforced PLA), and the comprehensive investigation of various process parameters effects on fatigue life and tensile strength. This type of research can contribute significantly to the understanding and optimization of composite thermoplastics fabricated by the FDM method, which has practical applications in industries such as aerospace, automotive, and consumer goods manufacturing.

## Experimental procedures

**Figure 1:**
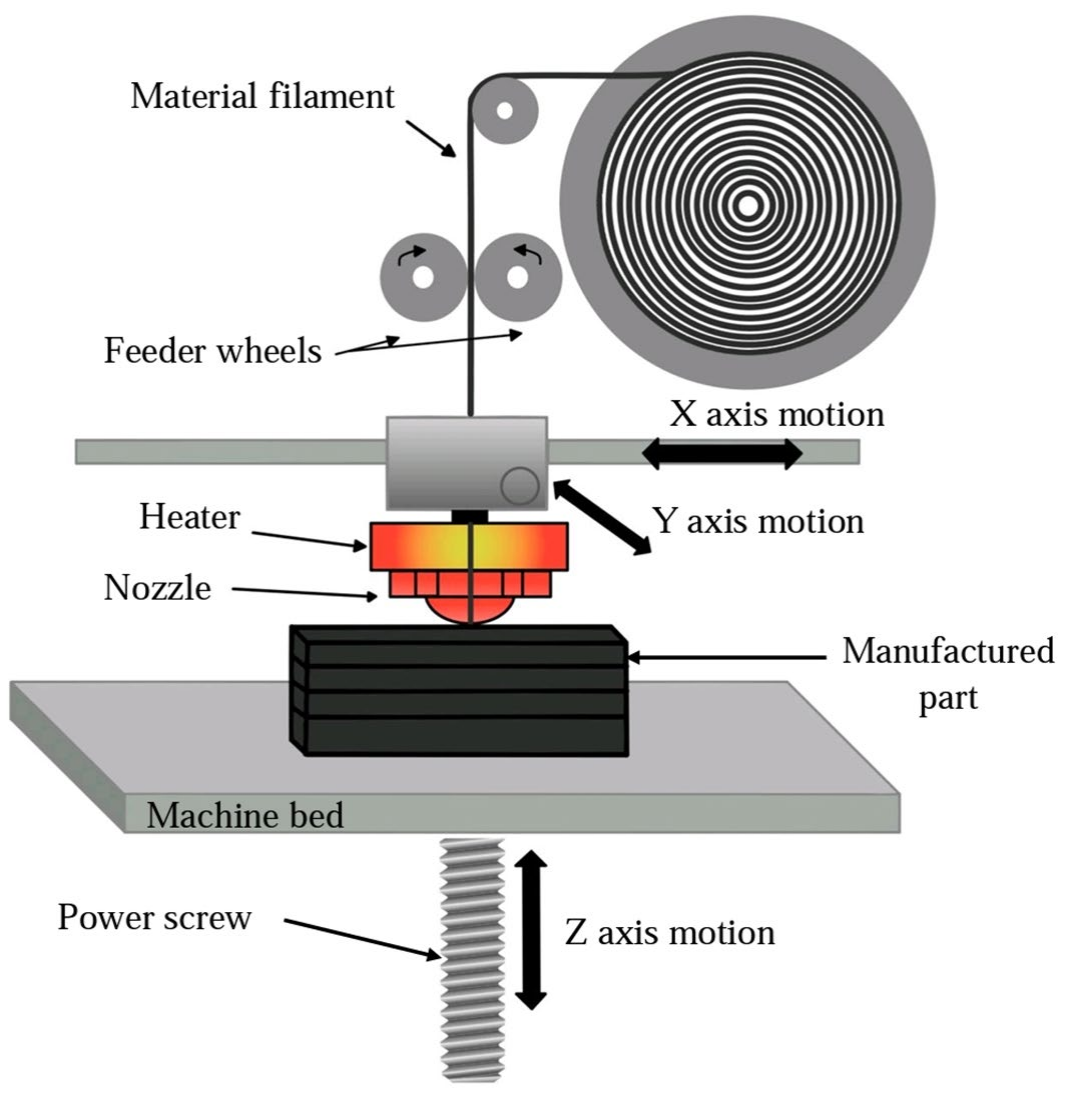
Material extrusion process. Figure was created by the authors with Adobe photoshop v. 21.2.11 software (URL: https://www.adobe.com/products/photoshop.html).

**Figure 2:**
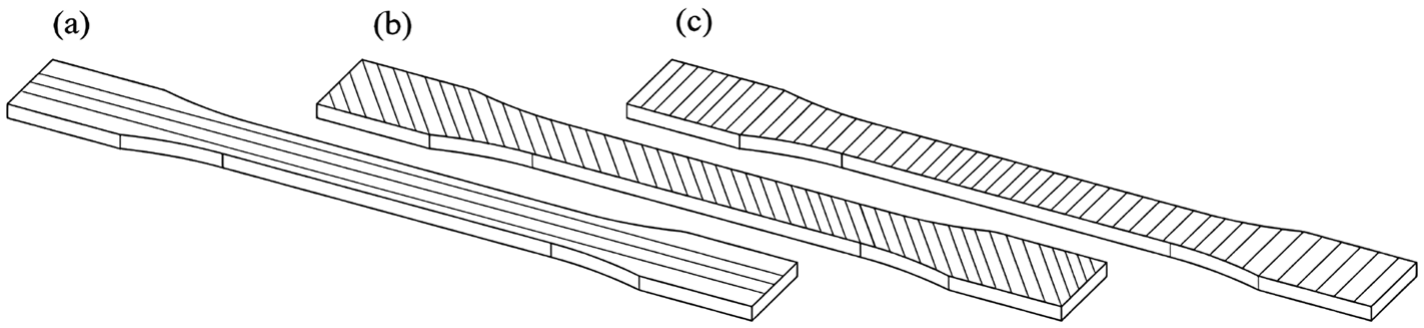
Schematic of raster angle (**a**) 90°, (**b**) 45° (**c**) 0°. Figure was created by the authors with SOLIDWORKS v21 software (URL: https://www.solidworks.com).

In FDM technology, thermoplastic is placed layer by layer in a predetermined path guided by a computer. Before forming, the thermoplastic was wrapped around a spool in the form of a string called the filament. The way the material extrusion process works is shown in Fig. 1, and it deposits the molded material layer by layer on the substrate^23^. Carbon fiber-based PLA filament with a diameter of 1.75 mm, a prevalent choice for home and commercial 3D printing, was used. The manufacturer added carbon fiber in powder form, constituting a precise 30% of the filament mixture, to enhance the thermoplastic's strength^24^. A steel nozzle was used in the construction of tensile and fatigue test samples. Hardened steel nozzles are specifically designed for the precise deposition of heavy and abrasive materials, including reinforced compounds. Crafted meticulously from top-grade steel, these nozzles exhibit exceptional hardness and remarkable resistance to wear and tear, thereby enabling prolonged and uninterrupted printing sessions. The printing temperature was 215 °C and the bed temperature was 60 °C. The layer height was chosen to be 0.2 mm^25^. The moderate printing speed was 40 mm/s, and besides checking the effect of the raster angle, two outer walls were stretched. The thickness of the shell was equal to the diameter of the nozzle^26–33^. All these parameters were selected based on the outcomes outlined in the references. The schematic of the raster angle is shown in Fig. 2.

### Tensile test and specimen

**Figure 3:**
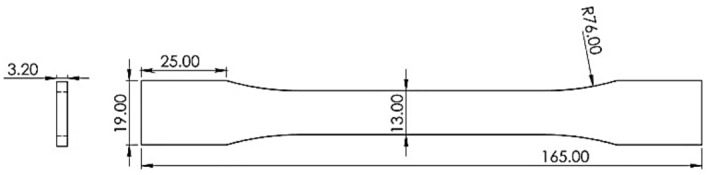
The geometry of the tensile test specimen. Figure was created by the authors with SOLIDWORKS v21 software (URL: https://www.solidworks.com).

**Figure 4:**
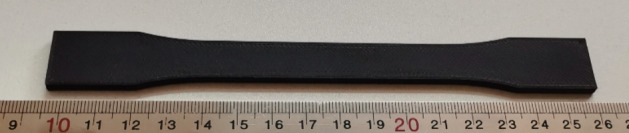
3D printed carbon fibers reinforced PLA tensile specimen. Photo was taken by the authors.

**Figure 5:**
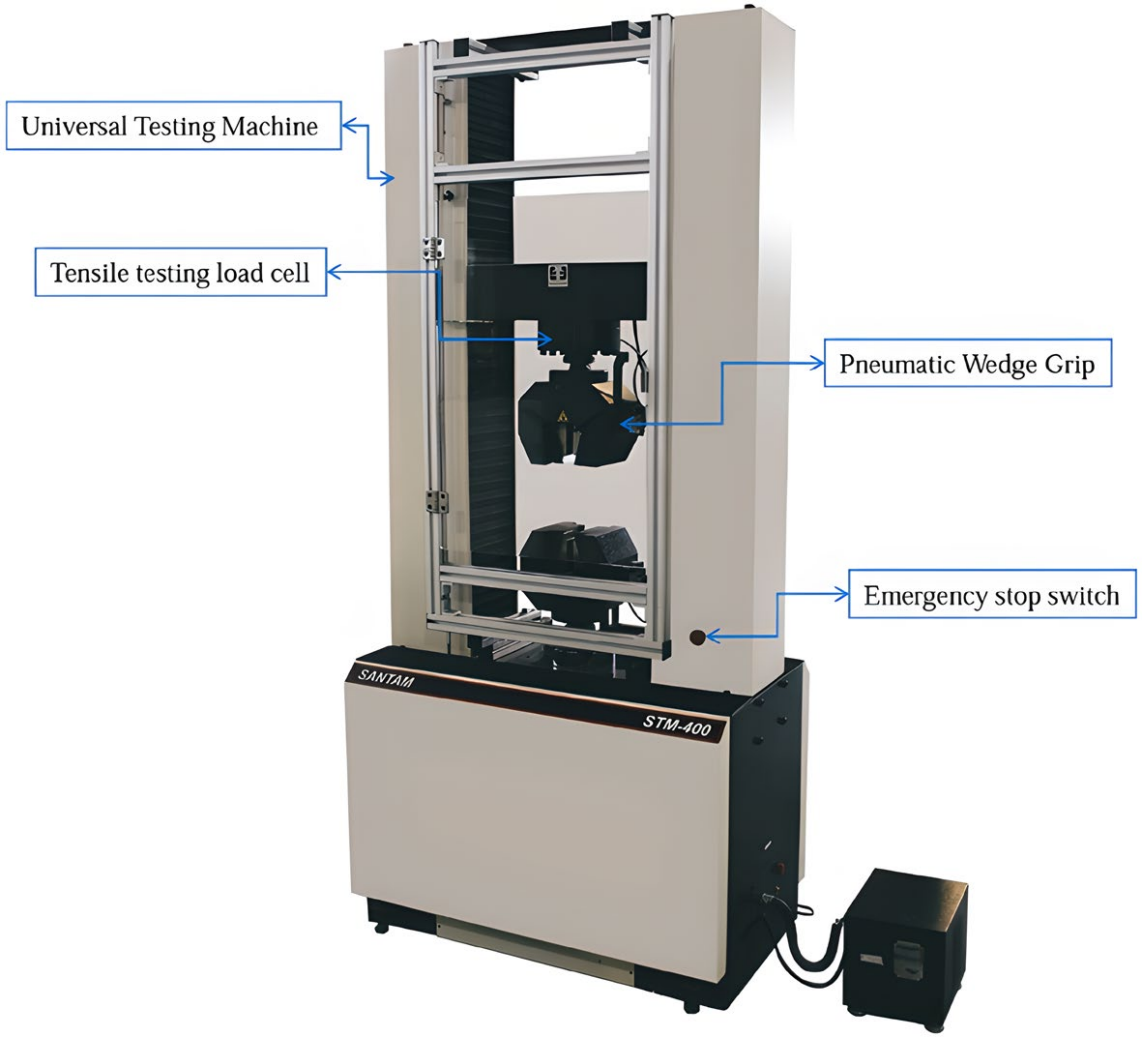
SANTAM STM-400 tensile testing machine. Image was taken by the authors and edited by Microsoft PowerPoint v2019 software (URL: https://www.microsoft.com/en-us/microsoft-365/powerpoint).

Tensile strength test samples were designed and made in the form of dumbbells based on the ASTM D638 standard. The dimensions of the standard specimen are shown in Fig. 3^34^. The tensile test design was carried out by the Taguchi L27 method. Layer thickness, nozzle diameter, angle of the raster, and infill density were the investigated parameters. The shell was composed with a + 45/− 45 direction. The printed sample of carbon fibers reinforced PLA tensile test is shown in Fig. 4. Validation of the tensile test was done by repeatability. The experimental design is shown in Table 1. STM-400 SANTAM universal machine was used for the tensile test, as shown in Fig. 5. The load cell of the tensile machine is adjustable and has a maximum load cell of 40 Tons. The load cell of the tension device is adjustable and has an utmost load cell of 400 KN. An extensometer was affixed to the specimens to enhance the accuracy of strain measurements. In this research, the EHR-10 short course extensometer was employed for measuring strain. The speed of the test was 5 mm per minute according to the standard as well as the test was at room temperature (25 °C)^35^.

**Table 1:** Taguchi L27 design for tensile testing.

Run	Nozzle diameter	Raster width	Infill Density	Raster angle
1	0.6	0.2	100	0
2	0.6	0.2	100	0
3	0.6	0.2	100	0
4	0.4	0.3	75	0
5	0.4	0.3	75	0
6	0.4	0.3	75	0
7	0.5	0.4	50	0
8	0.5	0.4	50	0
9	0.5	0.4	50	0
10	0.5	0.3	100	45
11	0.5	0.3	100	45
12	0.5	0.3	100	45
13	0.6	0.4	75	45
14	0.6	0.4	75	45
15	0.6	0.4	75	45
16	0.4	0.2	50	45
17	0.4	0.2	50	45
18	0.4	0.2	50	45
19	0.4	0.4	100	90
20	0.4	0.4	100	90
21	0.4	0.4	100	90
22	0.5	0.2	75	90
23	0.5	0.2	75	90
24	0.5	0.2	75	90
25	0.6	0.3	50	90
26	0.6	0.3	50	90
27	0.6	0.3	50	90

### Fatigue test and specimen

**Figure 6:**
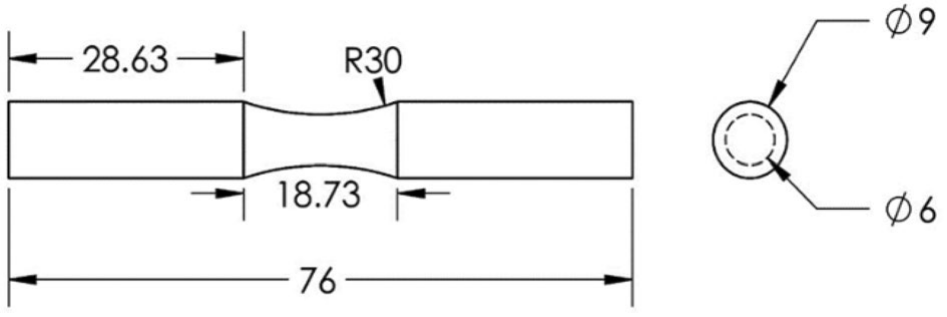
Standard dimensions of the fatigue test specimen. Figure was created by the authors with SOLIDWORKS v21 software (URL: https://www.solidworks.com).

**Figure 7:**
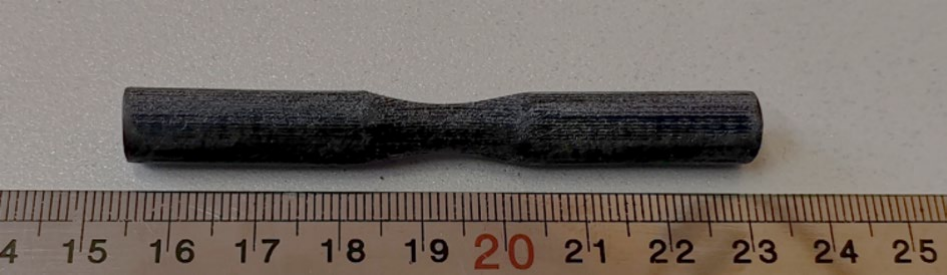
3D printed carbon fibers reinforced PLA fatigue specimen. Photo was taken by the authors.

**Figure 8:**
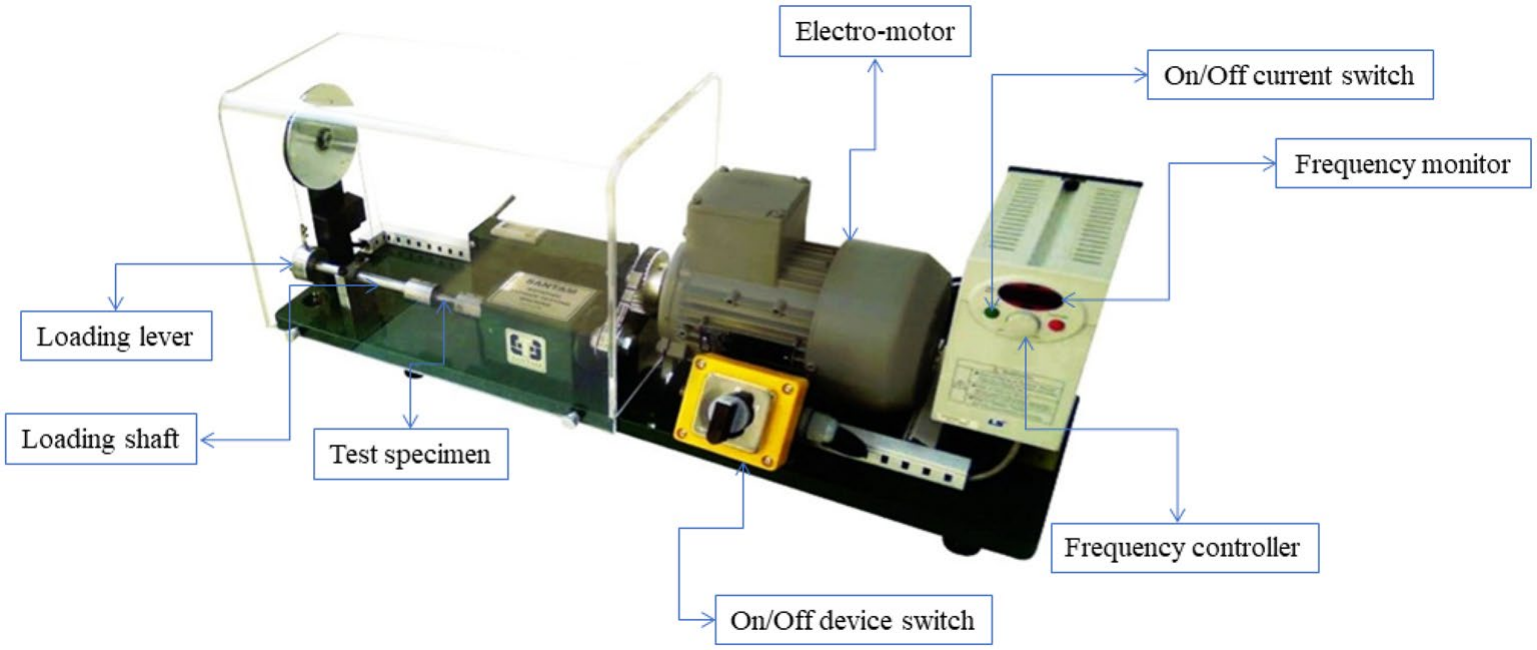
Rotary bending high cycle fatigue testing device. Image was taken by the authors and edited by Microsoft PowerPoint v2019 software (URL: https://www.microsoft.com/en-us/microsoft-365/powerpoint).

Dog-bone fatigue specimens, with a length of 76 mm, a holder diameter of 9 mm, and a gauge diameter of 6 mm, were fabricated according to the standard for fatigue testing. The standard dimensions of the fatigue sample are shown in Fig. 6. Raster angle of 90°, 45°, and 0° was investigated under cyclic bending loading. The infill density was considered 75 and 50%^31^. The printed fatigue sample of carbon fibers reinforced PLA is shown in Fig. 7^36^. Santam SFT-600 machines were used to find the life fatigue. This device is designed to apply a fully reversed (R = − 1) bending load and control stress in High cycle fatigue regime^37^. The load application rate and temperature were the same for all samples. The loading frequency of the bending fatigue test was 100 Hz and at room temperature (25 °C). The specimen was clamped between the two fixtures of the machine by 6 Allen screws, as shown in Fig. 8.

Knowing the material's UTS was effective for determining the applied stress. Different raster angles had different UTS. The samples with an infill density of 100% were used to determine UTS. The first stress level was 0.29UTS for the 0° sample, 0.34UTS for the 45° sample, and 0.52UTS for the 90° sample. For comparison, the stress levels were close together^38^.

Equation (1) was used to extract fatigue parameters from the test results^39^.

1 $$\sigma_{a} = \sigma^{\prime}_{f} \left( {2N_{f} } \right)^{b}$$

where ${\sigma }_{a}$ is the stress applied to the carbon fibers reinforced PLA and ${N}_{f}$ represents the fatigue life in the equation. The fatigue strength exponent is by parameter b, and ${\sigma {\prime}}_{f}$ is the fatigue strength coefficient.

### Data analysis

Minitab software was used to analyze the data and the influence of the examined factors on strength and life. Tensile strength data were tested and analyzed in 4 factors: layer thickness, nozzle diameter, ANGLE OF Raster, and infill density. Then the investigated parameters for the fatigue test were selected and analyzed according to the tensile test results.

## Results and discussion

### Mechanical properties

**Figure 9:**
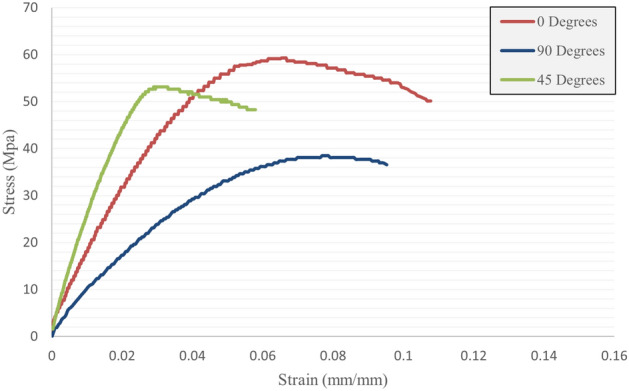
Stress–strain curve of carbon fibers reinforced PLA.

To evaluate the mechanical properties of carbon fiber-reinforced PLA, the effect of infill density, raster angle, nozzle diameter, and raster width were analyzed. The average stress–strain curve of three replicate samples with stuffed infill density is shown in Fig. 9. The results show that in the elastic region, carbon fiber-reinforced PLA follows Hooke's law and the applied stress is proportional to the strain of the material^40^. After the yield stress, it progressed to complete failure without strain hardening. The lower the raster angle of the layer in the tensile loading direction is, the stronger the material increases. The highest tensile strength was 59.29 Mpa at 0°, 53.16 Mpa at 45°, and 38.52 Mpa at 90°. Though the final strain at the time of failure does not completely follow the rule of reducing the raster angle and the lowest strain was obtained in the direction of 45°^41^.

**Figure 10:**
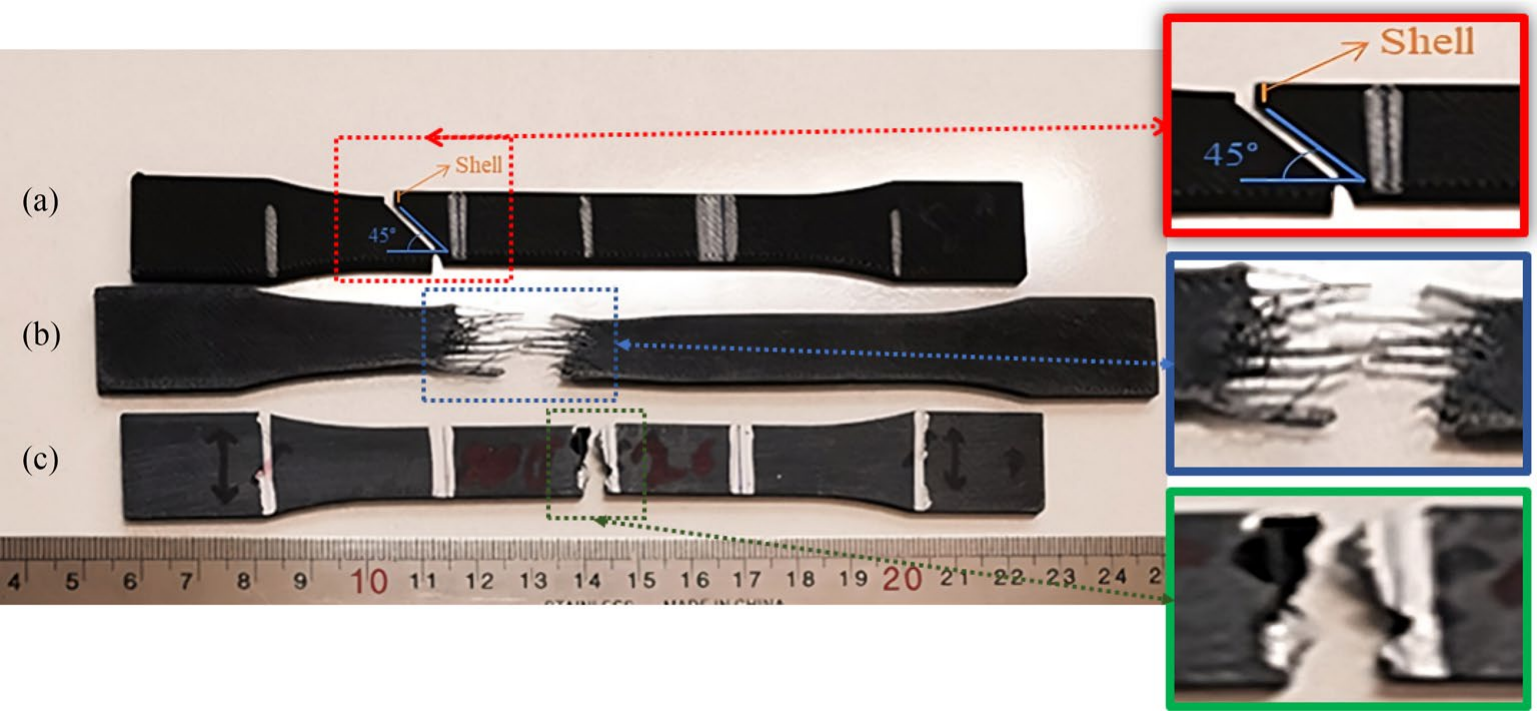
Carbon fibers reinforced PLA after tensile test (**a**) 45° (**b**) 0° (**c**) 90°. Image was taken by the authors and edited by Microsoft PowerPoint v2019 software (URL: https://www.microsoft.com/en-us/microsoft-365/powerpoint).

The tensile samples after the test are shown in Fig. 10. The samples reached their ultimate tensile strength until the rupture occurred in the shell. Moreover, the final failure occurred after the complete failure of the inner layers^42^. The sample with a layer deposition angle of 0° exhibited the highest degree of strain after reaching its ultimate strength, while the sample with a raster angle of 45° demonstrated the lowest degree of strain. The tensile data shows a positive correlation between the tensile strength of the samples and the reduction of the checker angle. As shown in Fig. 10, the catastrophic failure of each specimen occurred at the corresponding lattice angle. Notably, the delamination of the samples with a 0° grid angle was attributed to the weak bond between the carbon fibers and the matrix^43^.

**Figure 11:**
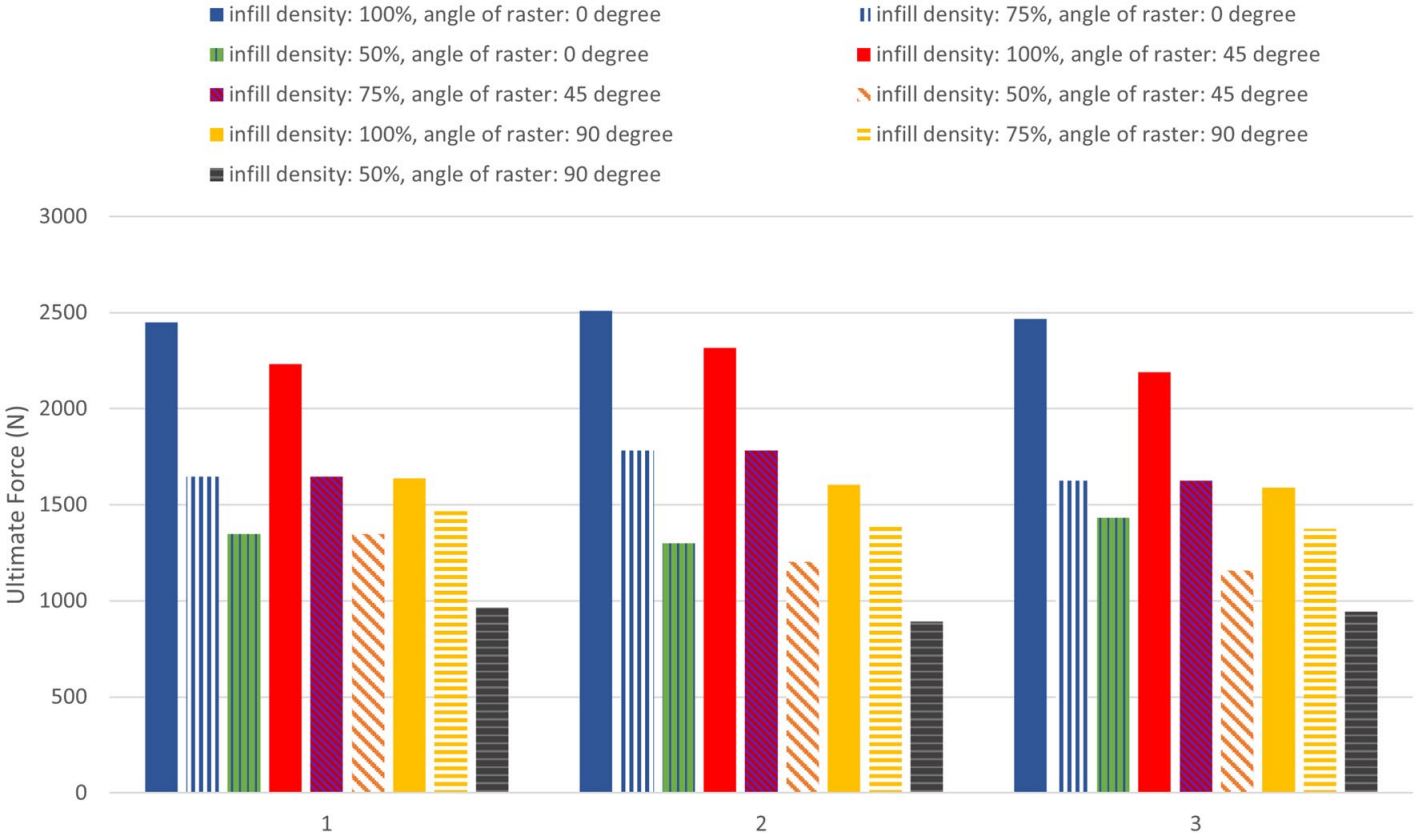
Maximum force applied in tensile loading on carbon fibers reinforced PLA.

Due to the anisotropic nature of the cross-sectional area of the samples with 50% and 75% porosity, the highest applied force and elongation were compared. Figure 11 illustrates the results of the maximum applied force versus the repetition number. The results indicate that, in addition to the layer deposition angle, the porosity percentage also has a significant impact on the material strength. The maximum applied force was observed for the PLA carbon fiber composite with 100% porosity, while the minimum applied force was observed for the 50% porosity composite. Moreover, the highest strength was observed for the 0-degree layer deposition angle sample, while the lowest applied force was associated with the 90-degree sample for the 75% and 50% porosity composites, similar to the 100% porosity composite^44^.

**Figure 12:**
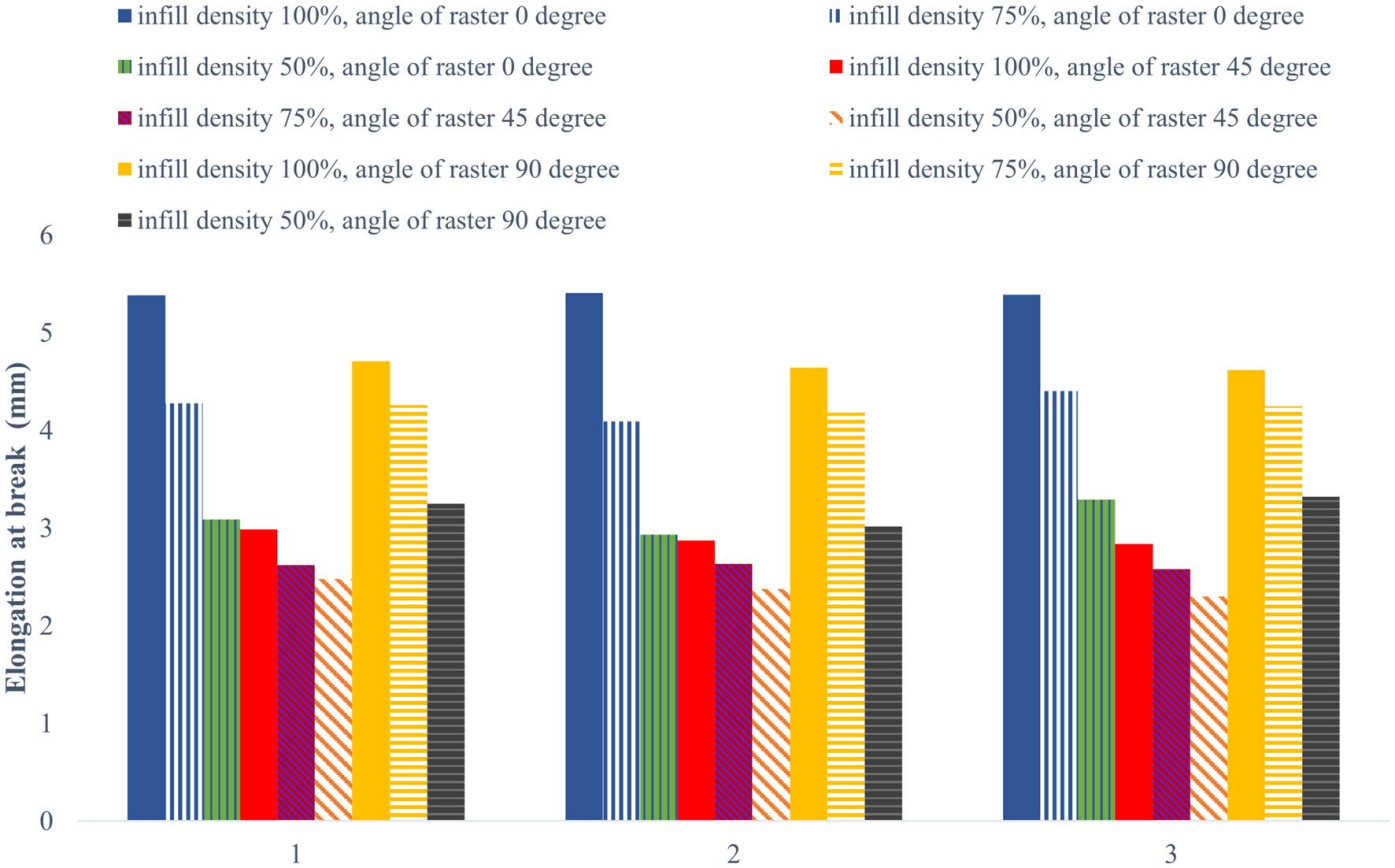
Elongation at the break under tensile loading of carbon fibers reinforced PLA.

The increase in elongation at break is shown in Fig. 12 by considering three repetitions of the diagram. Samples with 50% infill had the lowest elongation compared to the filling percentage. Additionally, the elongation at break exhibited varying results about the checkerboard angle. Samples with a 45° angle had lower elongation at the break than those with other angles. Moreover, the highest elongation at break was observed at the raster angle of 0 degrees. The tensile test was successful with no failed samples being detected^45^.

In this experiment, the width of the raster and the diameter of the nozzles were given significant consideration as they were found to have a substantial impact on the strength of the samples. By combining these parameters, the quality of the samples could be significantly improved, leading to an overall increase in their strength. To conduct a more accurate analysis of the effects of these parameters, the researchers utilized the MINITAB software. ANOVA and Signal to Noise Ratio analyses were conducted to identify the optimal combination of process parameters with precision and confidence^46^. The response table for average data and S/N ratio is shown in Table 2. Rank shows the influence of the tested factors. In these results, the first rank is related to the infill density (delta = 5.07), then the raster angle (delta = 3.33), the third rank is related to the raster width (delta = 0.45), and finally the nozzle diameter (delta = 0.3). “Signal” to “noise” represents the desired and undesired value for the output response, respectively.

**Table 2:** Mean response and S/N ratio for maximum applied force data.

Level/factors	Raster angle		Infill density		raster width		Nozzle diameter	
	S/N	Mean	S/N	Mean	S/N	Mean	S/N	Mean
1	65.50	1942	61.28	1177	64.22	1709	63.97	1612
2	64.44	1722	64.49	1696	64.13	1723	64.22	1673
3	62.17	1319	66.34	2111	63.77	1552	63.92	1698
Delta	3.33	622	5.07	934	0.45	171	0.30	86
Rank	2	2	1	1	3	3	4	4

**Figure 13:**
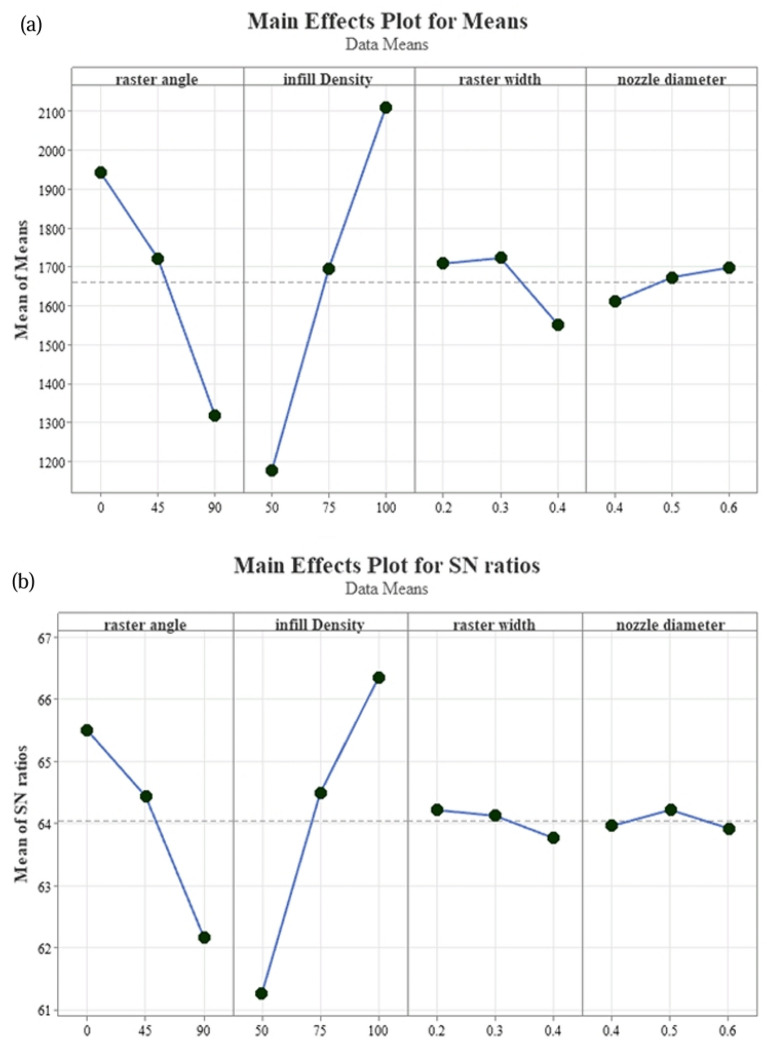
Main Effects Plot of Ultimate Force (N) obtained from Minitab: (**a**) for means values and (**b**) for signal-to noise ratios (S/N).

The parameters were compared in three levels, the effect of which is shown in Fig. 13. From these results, it can be seen that the nozzle diameter of 0.6 bears the highest and 0.4 the lowest force. Also, with raster width, 0.3 had the highest strength and 0.4 had the lowest strength. As the infill density decreases, the tensile strength decreases. As shown in Fig. 13. The angle in the loading direction can gain the utmost strength. The sharp slope of the line of these two parameters shows that the change in the parameters of the infill density and the raster angle creates substantial changes in the strength of the printed sample^47^. Based on the results obtained from Fig. 13a and b, it can be inferred that the highest strength is achieved by employing a raster angle of 0°, an infill density of 100%, a nozzle diameter of 0.6 mm, and a raster thickness of 0.3 mm. Conversely, the weakest performance can be attributed to the selection of parameters such as a raster angle of 90°, an infill density of 50%, a nozzle diameter of 0.4 mm, and a raster thickness of 0.4 mm.

**Figure 14:**
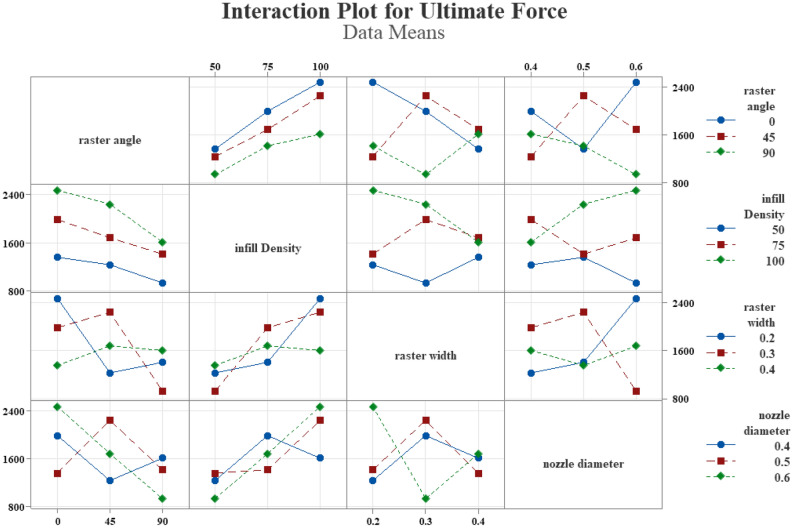
Interaction plots of inputs effects on the Ultimate Force.

The interaction between nozzle diameter, raster width, infill density, and raster angle was investigated. The impact of these parameters on the force was analyzed and presented in Fig. 14. Notably, no intersection line was found between the raster angle and infill density, as shown in Fig. 14. However, the absence of other parallel lines indicates that the fitted model may not be suitable for examining the interaction effect of the parameters on the force. Therefore, after the linear interaction model, a response surface methodology (RSM) was used to construct a multi-objective function for force^48^.

The response surface regression analyzes the correlation between variables, which determines the relationship between force and the parameters under investigation. Discerning the most significant correlation between factors, optimal responses can be attained. The linear interaction model is then utilized with the optimal parameter levels obtained from the response surface methodology. The regression model is shown in Eq. (2).

2 $${\text{Force}} = - {1}0{91} + {\text{2254A}} + {\text{4766B}} + {31}.{\text{26C}} - {2}.{\text{83D}} - {\text{1821A}}*{\text{A}} - {\text{9249B}}*{\text{B}} - 0.0{\text{838C}}*{\text{C}} - 0.0{\text{454D}}*{\text{D}}$$

The nozzle diameter, raster width, infill density, and raster angle are represented by A, B, C, and D, respectively. The correlation level is indicated by R-squared. In the software MINITAB, this level was calculated to be 98.80 for the fitted model, indicating a high level of correlation^49^.

**Figure 15:**
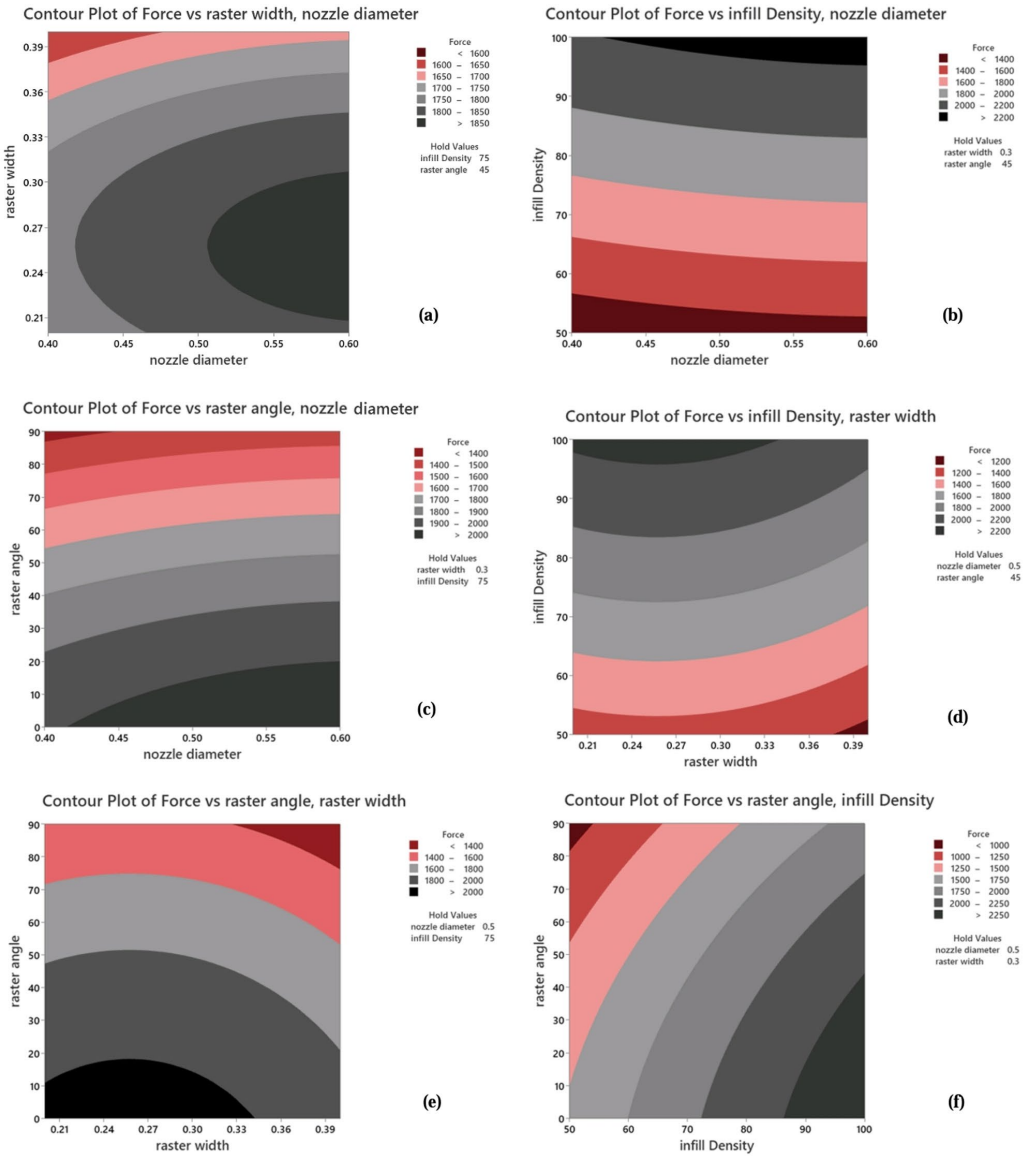
Depicts contour plots for Ultimate Force: (**a**) with a fixed infill density and raster angle, (**b**) with a fixed raster width and raster angle, (**c**) with a fixed infill density and raster width, (**d**) with a fixed nozzle diameter and raster angle, (**e**) with a fixed infill density and nozzle diameter, and (**f**) with a fixed nozzle diameter and raster width.

**Figure 16:**
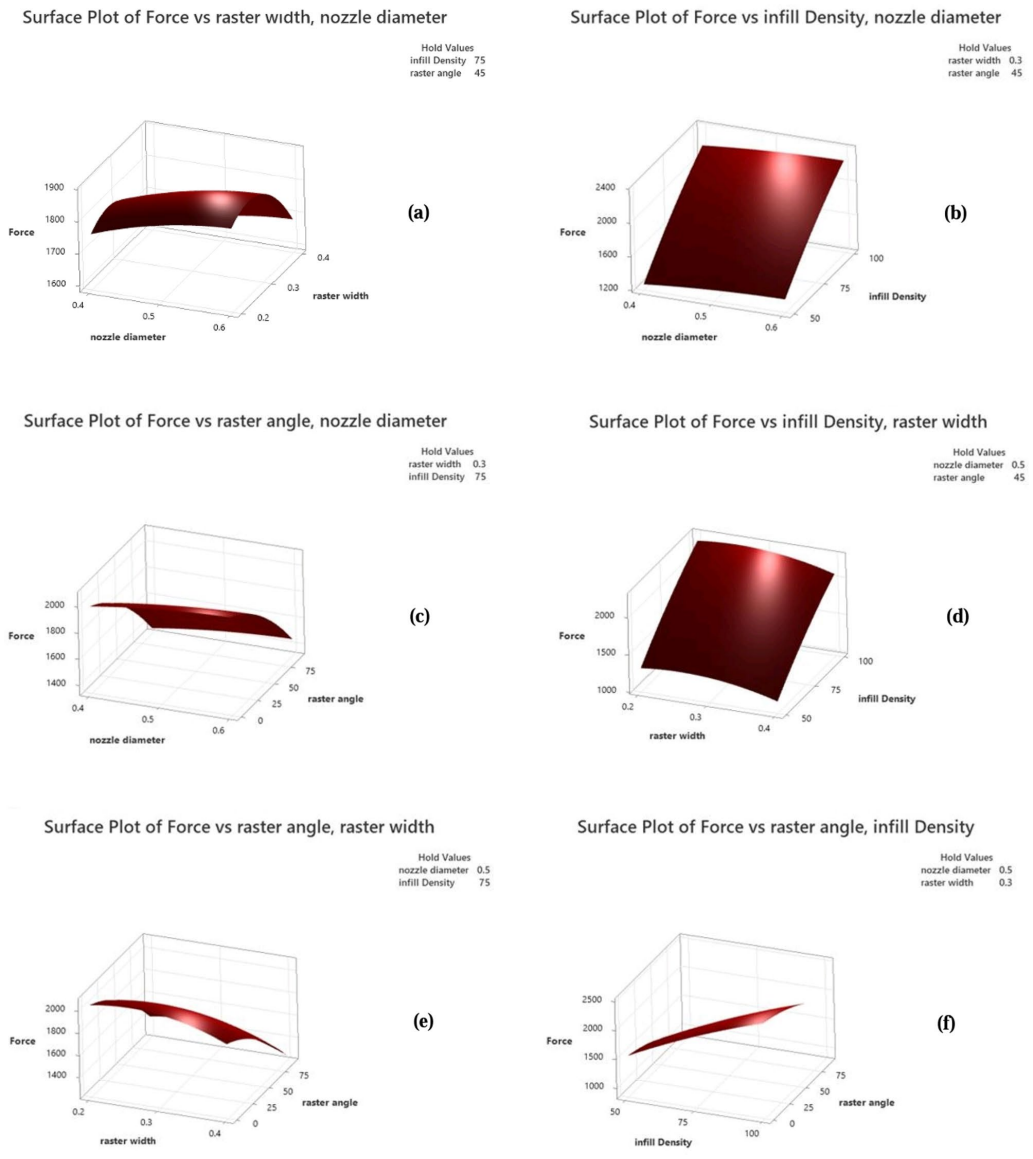
Depicts surface plots for Ultimate Force, (**a**) with a fixed infill density and raster angle, (**b**) with a fixed raster width and raster angle, (**c**) with a fixed infill density and raster width, (**d**) with a fixed nozzle diameter and raster angle, (**e**) with a fixed infill density and nozzle diameter, and (**f**) with a fixed nozzle diameter and raster width.

Contour plots are suitable for illustrating the desired response, indicating the ratio of two factors while the other factor is held at half its value. Graphical images are shown in Figs. 15 and 16 in the form of contour plots and surfaces. By comparing the contour and surface plots of the two factors, it can be observed that the optimal state occurs at a raster width ranging from 0.2 mm to 0.3 mm, a nozzle diameter between 0.5 mm to 0.6 mm, an infill density of 75% to 100%, and raster angles of 45° and 0°.

The comparison of tensile data from our current study with the research conducted by Kumar et al.^22^ and Rao et al.^21^ reveals an intriguing trend. It becomes evident that when it comes to particulate composites fabricated using the Fused Deposition Modeling (FDM) method, there is a noticeable increase in strength. However, it's essential to note that in direct comparison to continuous composites^10^, the tensile strength of particulate composites does exhibit a somewhat lower performance. This observation underscores the nuanced differences between these two types of composites, with each demonstrating its unique set of advantages and limitations.

### Fatigue behavior

**Figure 17:**
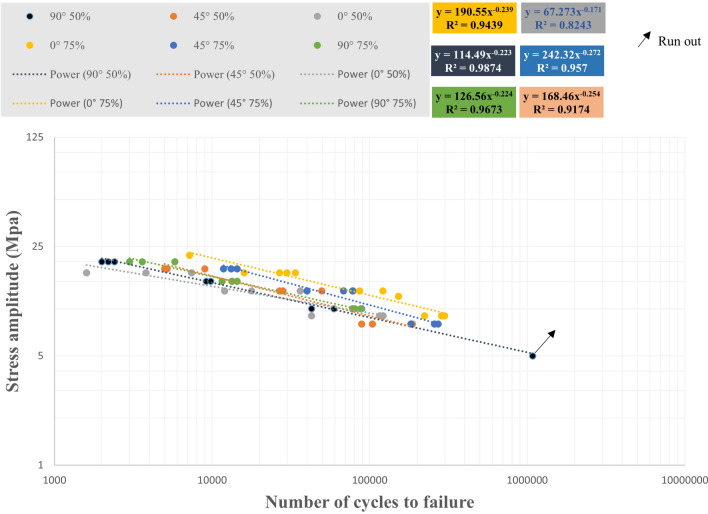
The S–N curve for all fatigue test data of carbon fibers reinforced PLA.

**Figure 18:**
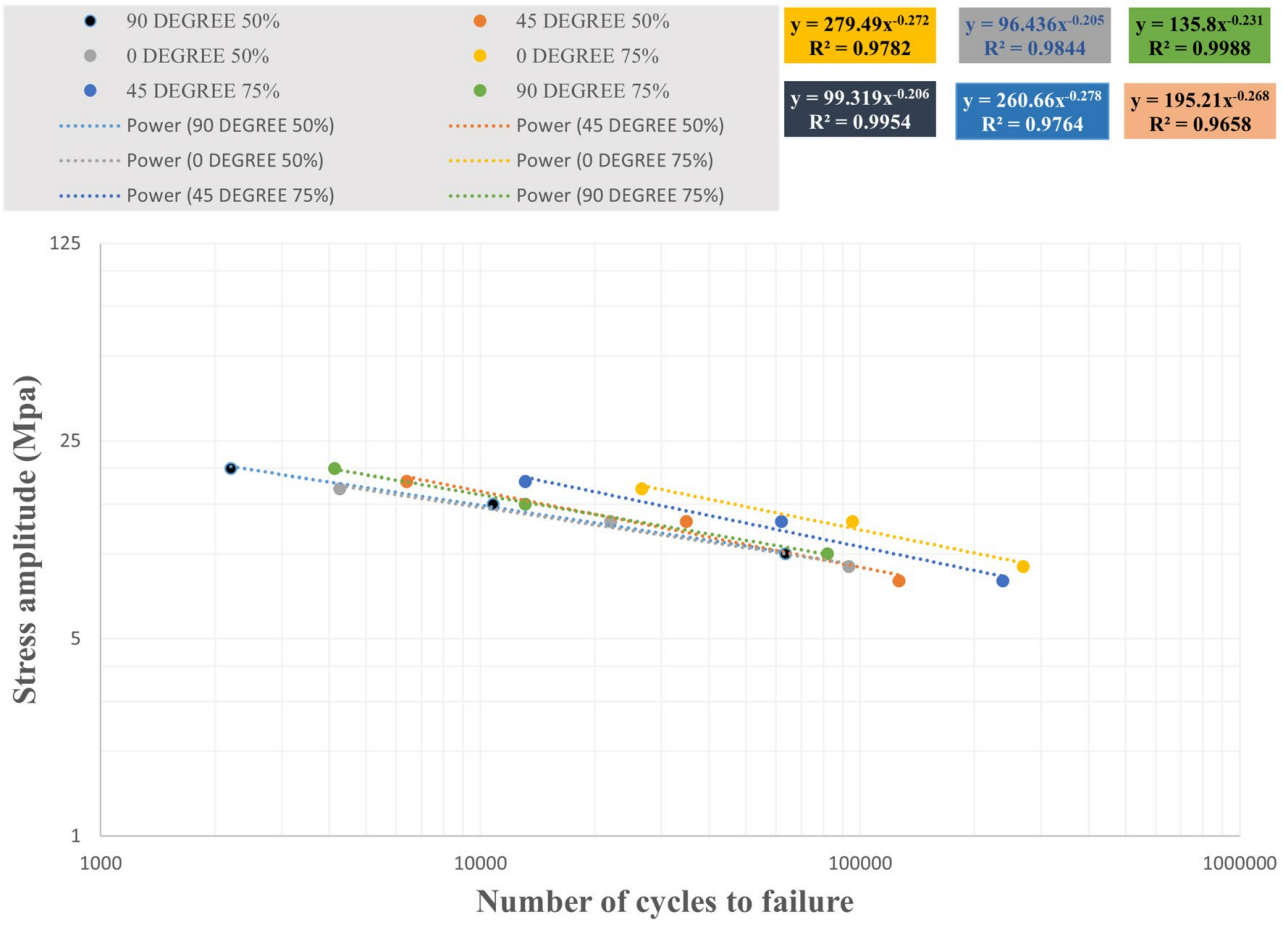
The S–N curve for the average fatigue test data of carbon fibers reinforced PLA.

The effect of 75% and 50% infill density on the fatigue life of three raster angle carbon fiber-reinforced PLA specimens was investigated. The results of the cyclic fatigue test in terms of stress-life curves for all specimens and the mean life for the compared data are shown in Figs. 17 and 18. It can also be observed from Figs. 17 and 18 that infill density also affects the material life. The specimen life with 75% infill density in three directions was greater than that of specimens with 50% infill density.

Repeatability was set to three to ensure the reliability of each specimen's fatigue life and mean fatigue life. The presence of carbon fiber particles and voids in the gauge area resulted in the significant scatter of the fatigue life of specimens with 50% infill density. The type of layer structure and infill density directly affected the scatter band. The fatigue strength coefficient and exponent were extracted using R-squared correlation and presented in Table 3.

**Table 3:** The fatigue property parameters of carbon fibers reinforced PLA extracted from cyclic fatigue tests.

Infill density	Raster angle	All Data			Mean value		
		${R}^{2}$	${\sigma {\prime}}_{f}$	$b$	${R}^{2}$	${\sigma {\prime}}_{f}$	$b$
50%	90°	0.9874	114.49	− 0.223	0.9954	99.319	− 0.206
	45°	0.9174	168.46	− 0.254	0.9658	195.21	− 0.268
	0°	0.8243	67.273	− 0.171	0.9844	96.346	− 0.205
75%	90°	0.9673	126.56	− 0.224	0.9988	135.8	− 0.231
	45°	0.957	242.32	− 0.272	0.9764	260.66	− 0.278
	0°	0.9439	190.55	− 0.239	0.9782	279.49	− 0.272

The fatigue strength coefficient was higher for the 45° raster angle with 50% infill density than for other angles, according to Table 3. The fatigue strength at the 45° angle was also higher than other angles for data at 75% infill density. However, the mean data analysis showed the fatigue strength coefficient at the 0° angle was slightly higher than at other angles.

**Figure 19:**
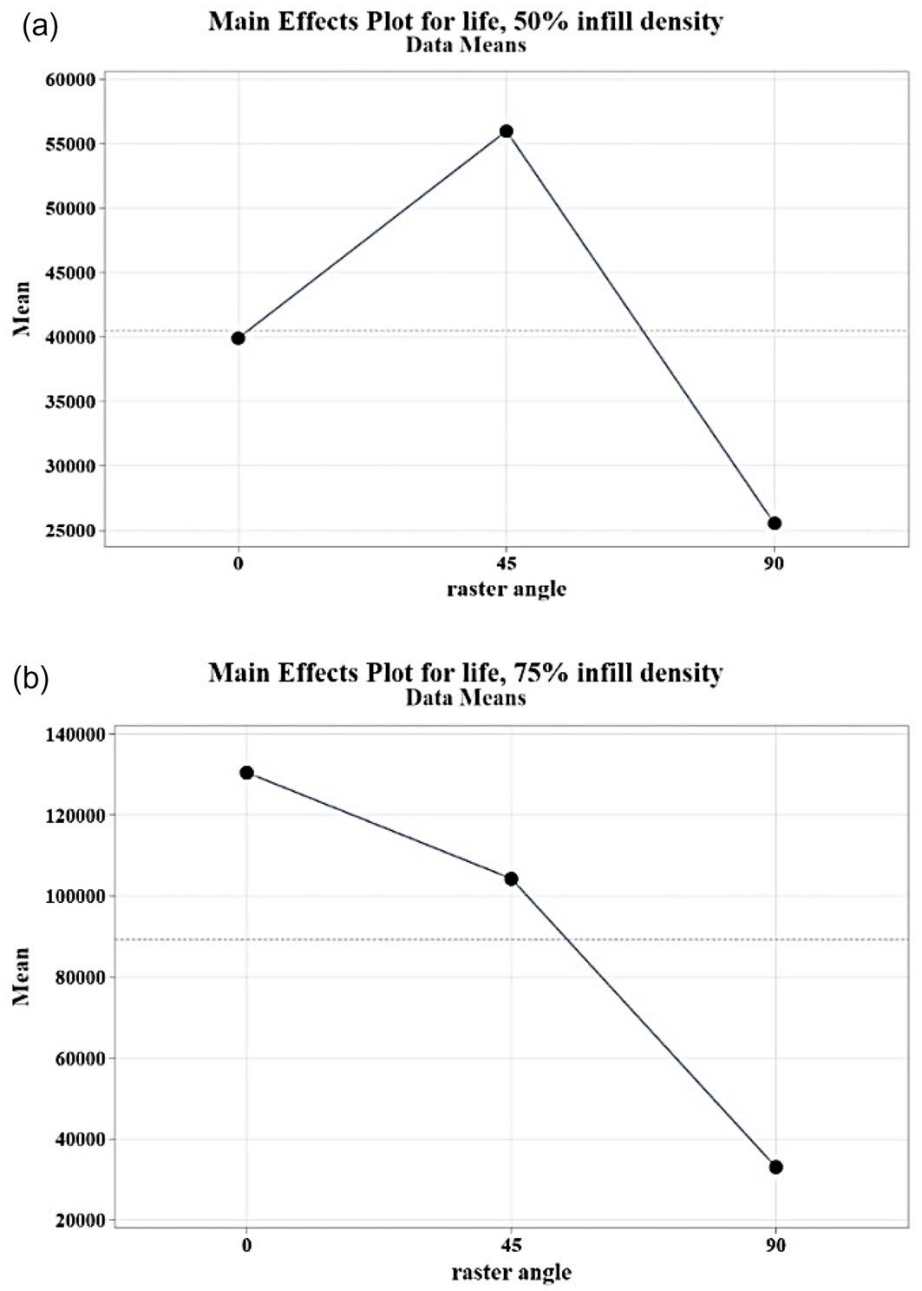
The main effect plot of fatigue life at (**a**) 50% and (**b**) 75% infill density.

The investigation of fatigue life at different infill density levels showed different behaviors. As demonstrated in Fig. 19, at 50% infill density, the mean fatigue life of specimens with a 45° raster angle is higher than that of specimens with a 0° angle. Conversely, at 75% infill density, the highest mean fatigue life was observed for specimens with a 0° raster angle. Additionally, specimens with a 90° raster angle had the lowest strength for both 50% and 75% infill density levels, As shown in Fig. 19b.

**Figure 20:**
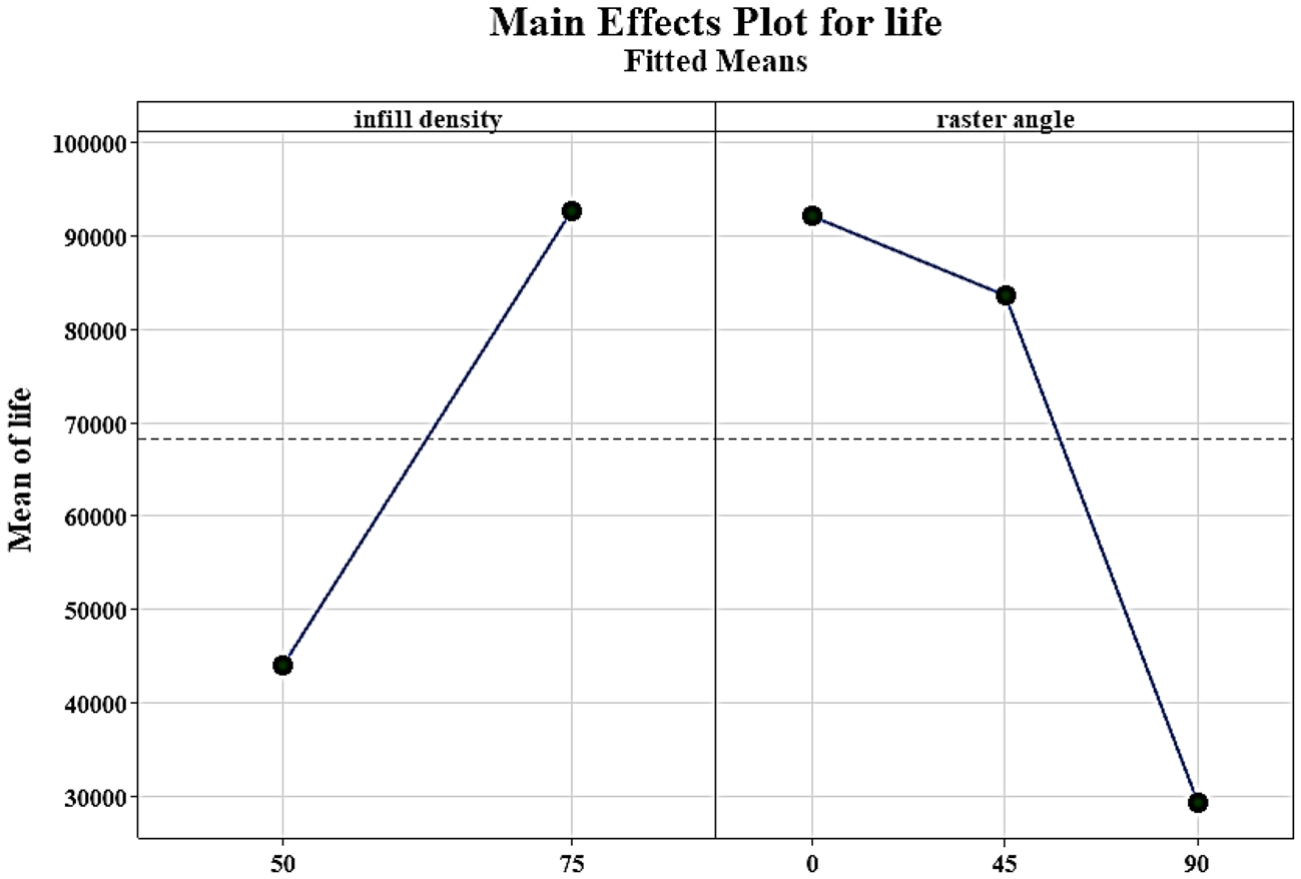
The main effect plot for the infill density and angle raster on the fatigue life of carbon fibers reinforced PLA.

The statistical analysis of factors affecting fatigue life is shown in Fig. 20. The results indicate a direct relationship between fatigue life and the increase in infill density. Moreover, specimens with a 0° raster angle had the highest fatigue life, while specimens with a 90° raster angle had the lowest strength^50^.

**Figure 21:**
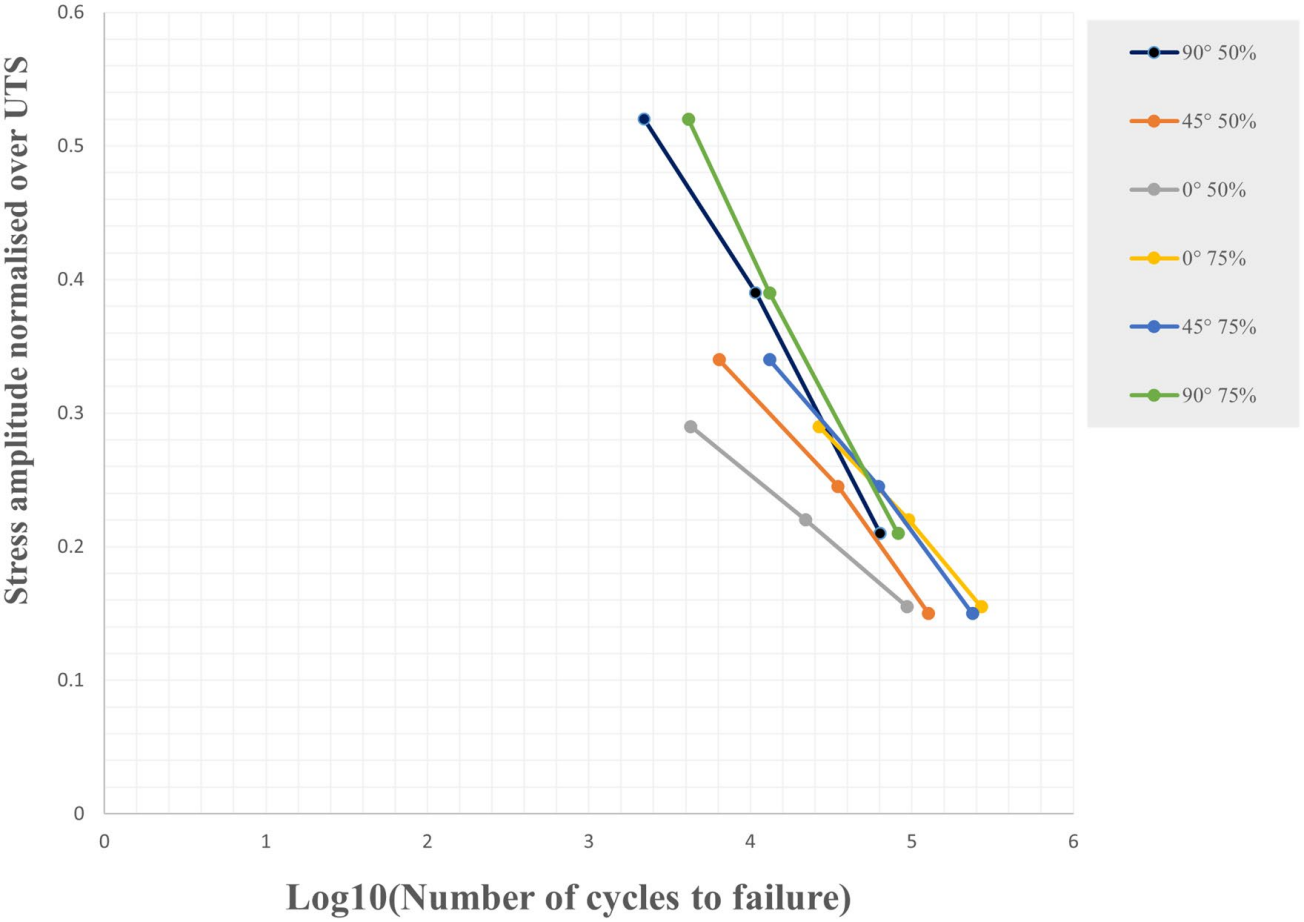
The S–N curve normalised over UTS of carbon fibers reinforced PLA.

The chosen fatigue stress levels were based on the Ultimate Tensile Strength of carbon fiber-reinforced PLA, which was printed with full infill density^51^. The results of the testing were normalized to the tensile test results and are shown in Fig. 21. The graph in Fig. 21 shows that the fatigue life of the carbon fiber-reinforced PLA increased by between 2 and 21% depending on the angle of the stress^52^. At a 0° angle, the fatigue life increased by 9% to 21%, making it the most favorable angle for the carbon fiber-reinforced PLA. At a 45° angle, the fatigue life increased by 5% to 8%, while at a 90° angle, it increased by 2% to 8%. These findings are significant as they demonstrate that carbon fiber-reinforced PLA with full infill density can withstand a range of stress levels without failing. Furthermore, the results provide insights into the most favorable angle for the carbon fiber-reinforced PLA, which can be useful when designing parts that will be subjected to fatigue loading.

## Conclusions

In this study, carbon fiber-reinforced PLA composite filaments were manufactured using fused deposition modeling to investigate tensile strength and fatigue. Parameters examined for tensile strength included infill density, raster angle, raster width, and nozzle diameter. Tensile strengths at 100% infill with layer deposition angles of 0, 45, and 90 degrees were 59.29 MPa, 53.16 MPa, and 39.52 MPa, respectively. Fatigue testing stress levels were also determined using samples with a 100% infill density. The impact of examined parameters on strength was determined based on the results of tensile testing and statistical analysis. Infill density, raster angle, raster width, and nozzle diameter had the highest to lowest impact on strength. Results showed that increased nozzle diameter and infill density led to increased strength, while decreased raster width and raster angle also resulted in increased strength. Optimal strength was achieved with a 0° angle, 100% infill density, 0.6 mm nozzle diameter, and 0.3 mm raster width. Optimal parameters for elongation at break and strength differed somewhat. The Tensile specimens reached UTS when the shell layers were completely separated. The raster angle had the greatest effect on deformation, with a 0° angle resulting in the highest amount of deformation and a 90° angle resulting in the lowest. Similarly, the highest elongation at break was observed at a 0° angle, while the lowest was observed at 90°. Fatigue testing was conducted at three stress levels to compare the impact of raster angle and infill density. The optimal effect of print parameters was observed at stress levels between 0.15UTS and 0.52UTS. Optimizing additive manufacturing parameters is essential for increasing material lifespan and withstanding stress close to UTS. Changes in infill density and angle significantly impact lifespan. Consideration of optimal parameters is crucial to achieving desired results. The highest strength was observed at a 45° raster angle with a 50% infill density, while at a 75% infill density, the highest strength was observed at a 0° raster angle, while the lowest was observed at 90°.

These findings highlight the role of FDM manufacturing parameters in improving fatigue life and tensile strength of carbon fibers reinforced PLA and provide guidance for using carbon fibers reinforced PLA 3D printing and increasing fatigue life and strength in industrial and domestic applications.

## Data Availability

The datasets generated during and/or analysed during the current study are available from the corresponding author on reasonable request.
